# Highly Selective and Sensitive Detection of Hg^2+^ Based on Förster Resonance Energy Transfer between CdSe Quantum Dots and g-C_3_N_4_ Nanosheets

**DOI:** 10.1186/s11671-018-2647-6

**Published:** 2018-08-13

**Authors:** Shan Wang, Ruiqing Liu, Chenchen Li

**Affiliations:** School of Chemistry and Chemical Engineering, Xianyang Normal University, Xianyang, 712000 People’s Republic of China

**Keywords:** FRET, CdSe QDs, g-C_3_N_4_ nanosheet, Sensor

## Abstract

In the presence of Hg^2+^, a fluorescence resonance energy transfer (FRET) system was constructed between CdSe quantum dots (QDs) (donor) and g-C_3_N_4_ (receptors). Nanocomposites of g-C_3_N_4_ supported by CdSe QDs (CdSe QDs/g-C_3_N_4_ nanosheets) were fabricated through an electrostatic interaction route in an aqueous solution. The nanocomposites were characterized by X-ray photoelectron spectroscopy, X-ray diffraction, Fourier-transform infrared spectroscopy, and transmission electron microscopy. Results showed that the g-C_3_N_4_ nanosheets were decorated randomly by CdSe QDs, with average diameter of approximately 7 nm. The feasibility of the FRET system as a sensor was demonstrated by Hg (II) detection in water. At pH 7, a linear relationship was observed between the fluorescence intensity and the concentration of Hg (II) (0–32 nmol/L), with a detection limit of 5.3 nmol/L. The new detection method was proven to be sensitive for detecting Hg^2+^ in water solutions. Moreover, the method showed high selectivity for Hg^2+^ over several metal ions, including Na^+^, Mg^2+^, Ca^2+^, Pb^2+^, Cr^3+^, Cd^2+^, Zn^2+^, and Cu^2+^. The CdSe QDs/g-C_3_N_4_ nanosheet conjugate exhibited desirable long-term stability and reversibility as a novel FRET sensor. The novel FRET-based fluorescence detection provided an attractive assay platform for quantifying Hg^2+^ in complex water solutions.

## Background

The main cause of mercury poisoning in humans was polluted natural waters [1]. Hg^2+^ ion metabolism by aquatic microbes produces methyl mercury, which was a potent neurotoxin associated with cognitive and motion disorders [2]. Therefore, mercury detection methods that are rapid, cost-effective, facile, and applicable to complex environments are necessary. Particularly, nanomaterials with unique optical properties can be employed to develop optical sensors with high sensitivity and selectivity [3]. Semiconductor quantum dots (QDs), fluorescent metal nanoclusters (NCs), noble metal nanoparticles (NPs), and carbon nanodots (CDs) were commonly used in the design of Hg^2+^ optical sensors because of their distinct properties, such as easy synthesis, high stability, functionalization, and biocompatibility. Many fluorescent sensors for Hg^2+^ had been reported [4–8]. For example, Huang et al. [9] developed a time-gated Förster resonance energy transfer (FRET) sensor for Hg^2+^ detection. Moreover, different FRET systems had been developed for the detection for Hg^2+^ [10–12]. Notably, FRET systems could be similarly built using nanoparticles, such as QDs, as well as organic and inorganic NPs [13–15]. Among the nanoparticles, g-C_3_N_4_ nanosheets had attracted widespread interest [16, 17]. Although g-C_3_N_4_ nanosheets have been applied as sensors, a FRET detection system with g-C_3_N_4_ nanosheets and CdSe QDs for metal ions has not been reported. FRET-based fluorescence sensing systems offer multiple advantages [18].

**Fig. 1:**
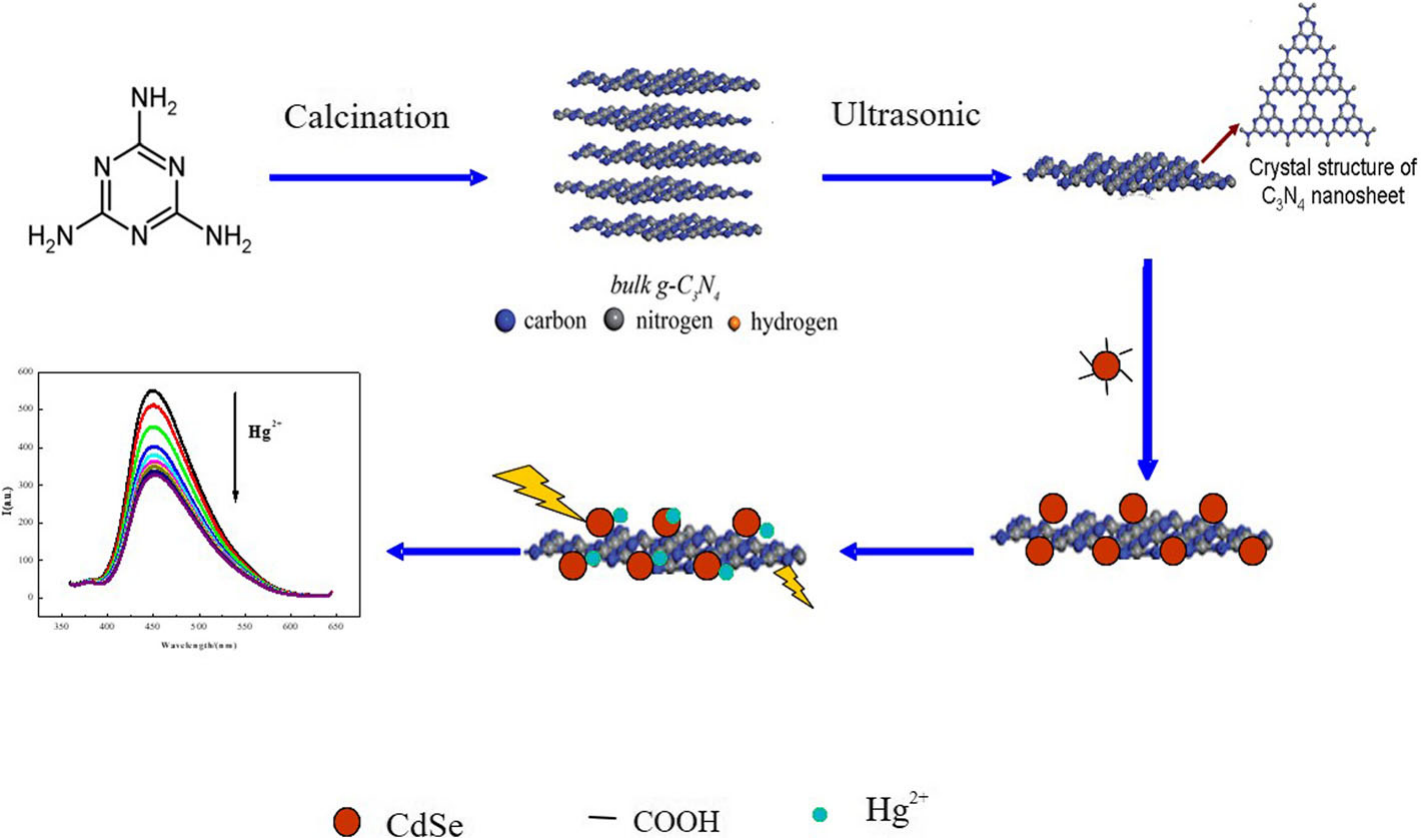
The mechanisms of FRET-based detection for mercury ions

In the present study, a new FRET-based fluorescence sensor was developed to detect mercury ions in aqueous media by using g-C_3_N_4_ nanosheets and CdSe QDs particles as vehicles. The proposed mechanism was illustrated in Fig. 1.

## Methods

### Materials

Mercury (II) chloride (HgCl_2_) was purchased from Tong Ren Chemical Research Institute (Guizhou, China). Urea and CdSe QDs were purchased from Aladdin Reagent Company (Shanghai, China). Other reagents and chemicals were of analytical reagent grade and used without further purification. All solutions were prepared using purified water from a Milli-Q gradient water purification system (Millipore Inc., USA; nominal resistivity 18.2 MÙ cm).

### Characterization

An X-ray diffractometer (Rigaku D/max-2400) was used to obtain diffraction patterns. Ultraviolet–visible (UV–vis) spectra were recorded on a UV–vis 800 spectrophotometer at room temperature. Fourier-transform infrared (FTIR) spectra were recorded on a Nicolet-nexus670 spectrometer using KBr. Fluorescence measurements were performed at room temperature with an RF-5301PC fluorescence spectrometer. X-ray photoelectron spectroscopy (XPS) measurements were performed using a multifunctional spectrometer (Thermo Scientific).

### Construction of FRET Sensor between the g-C_3_N_4_ Nanosheets and CdSe QD Particles

In a typical procedure, g-C_3_N_4_ (125 mg, which was synthesized according to our previous report [19]) was dispersed in 250 mL of water (1:1) and ultrasonicated for 5 h at ambient temperature. Then, CdSe QDs (1.838 g, 0.0216 mol) were dissolved in the solution by sonication for 2 h. Given that the amine group on the g-C_3_N_4_ nanosheets and CdSe QDs had a carboxyl group, g-C_3_N_4_ nanosheets and CdSe QDs nanoparticles would be combined by electrostatic interaction. All solutions were prepared in Milli-Q gradient water (pH = 7). The CdSe QDs/g-C_3_N_4_ nanosheet conjugate emission spectra were recorded. All samples were excited at 334 nm, which was near the minimal acceptor absorption.

### Fluorescence Detection of Hg^2+^

Hg^2+^ was quenched at room temperature in water. During a typical operation, 10 μL of the CdSe QDs/g-C_3_N_4_ nanosheet conjugates was added to 3 mL of ultrapure water, and then the calculated amount of Hg^2+^ was added. The emission spectra of the CdSe QDs/g-C_3_N_4_ nanosheet conjugates were recorded 2 mins later at room temperature.

### Interference and Competition Analyses

The response of the FRET nanoprobe to other metal ions (Na^+^, Mg^2+^, Ca^2+^, Pb^2+^, Cr^3+^, Cd^2+^, Zn^2+^, and Cu^2+^) was studied through fluorescence spectroscopy. Studies were carried out using the CdSe QDs/g-C_3_N_4_ nanosheet conjugates emitting at 450 nm. The conjugate solution was placed in a 1-cm optical path quartz fluorescence cuvette. Fluorescence intensity was measured at emission wavelength of 450 nm under excitation wavelength of 334 nm in the presence of each possible interference (32 nM). Competition assays were also performed for all the possible interferences previously analyzed. For competition experiments, 32 nM Hg^2+^ aqueous solutions were prepared.

## Results and Discussion

### Characterization

**Fig. 2:**
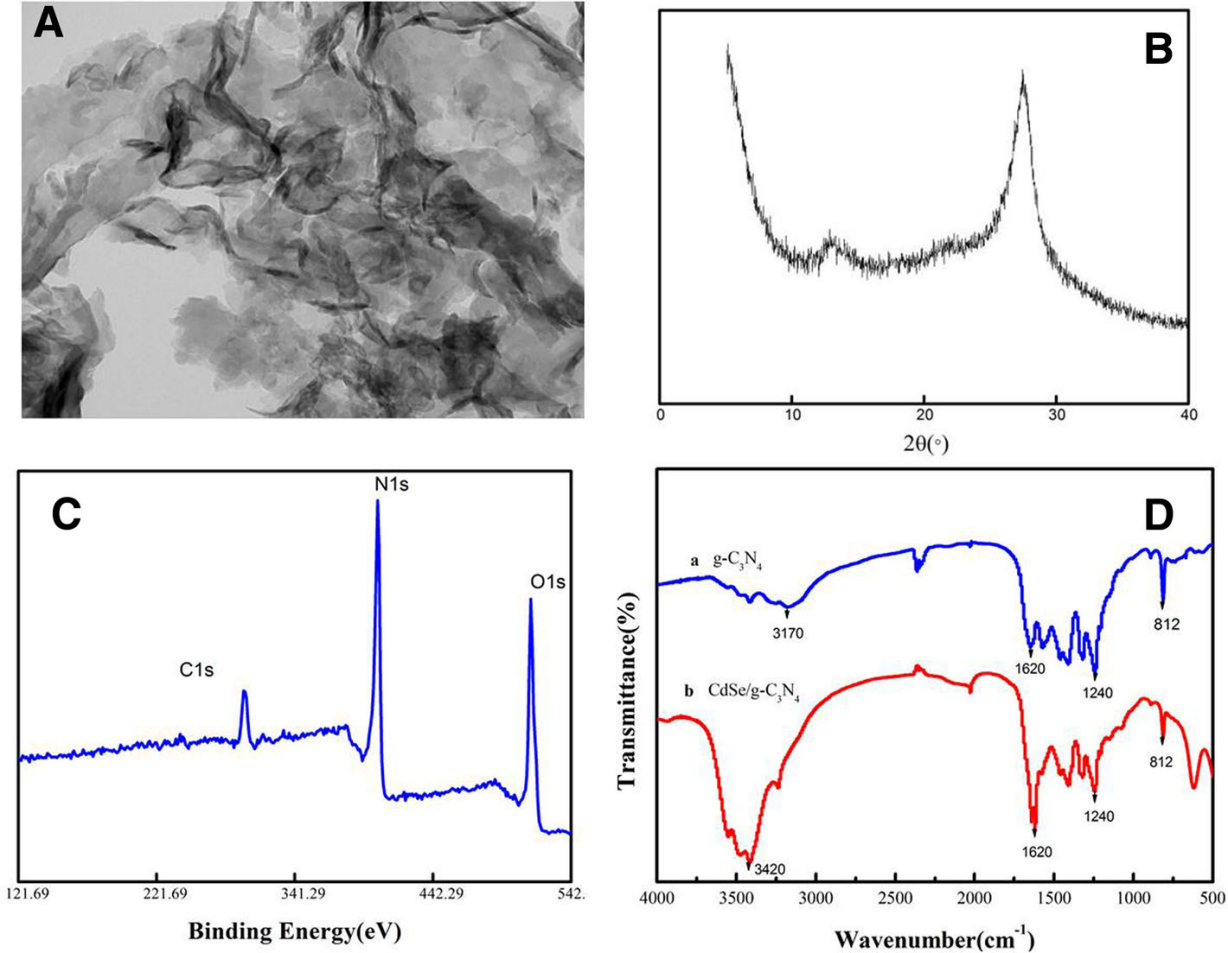
Characterization of the as-prepared g-C_3_N_4_ nanosheet. **a** TEM image. **b** XRD image. **c** XPS spectrum. **d** FTIR spectrum

The structure and morphology of g-C_3_N_4_ nanosheets were characterized by TEM, XPS, and XRD. The TEM image in Fig. 2a showed that the g-C_3_N_4_ nanosheet possessed a graphene-like morphology that mainly consists of a few layers [19]. Figure 2a showed the XRD patterns of the g-C_3_N_4_ nanosheets. The strong XRD peak centered at 27.4° corresponded to the typical graphitic interlayer stacking (002) peak of g-C_3_N_4_. The small peak at 13.1° corresponded to the periodic in-plain structural packing feature within the sheets [20, 21]. XPS measurement was used to analyze the valence states of g-C_3_N_4_ nanosheets. The XPS spectrum in Fig. 2c showed the C–C bonded to N at 284.8 and 288.0 eV, and the N 1 s spectrum was at 397.04 eV. In Fig. 2d, the peak at 811 cm^−1^ was attributed to the vibration of the triazine ring. The peaks around 1000 cm^−1^ represented the stretching modes of CN heterocycles, and the peak at 1800 cm^−1^ corresponded to C–NH–C. The peaks at 300–3600 cm^−1^ corresponded to N–H and O–H stretching vibrations [22].

### UV-vis and Fluorescence Properties of CdSe QDs/g-C_3_N_4_ Nanosheets

**Fig. 3:**
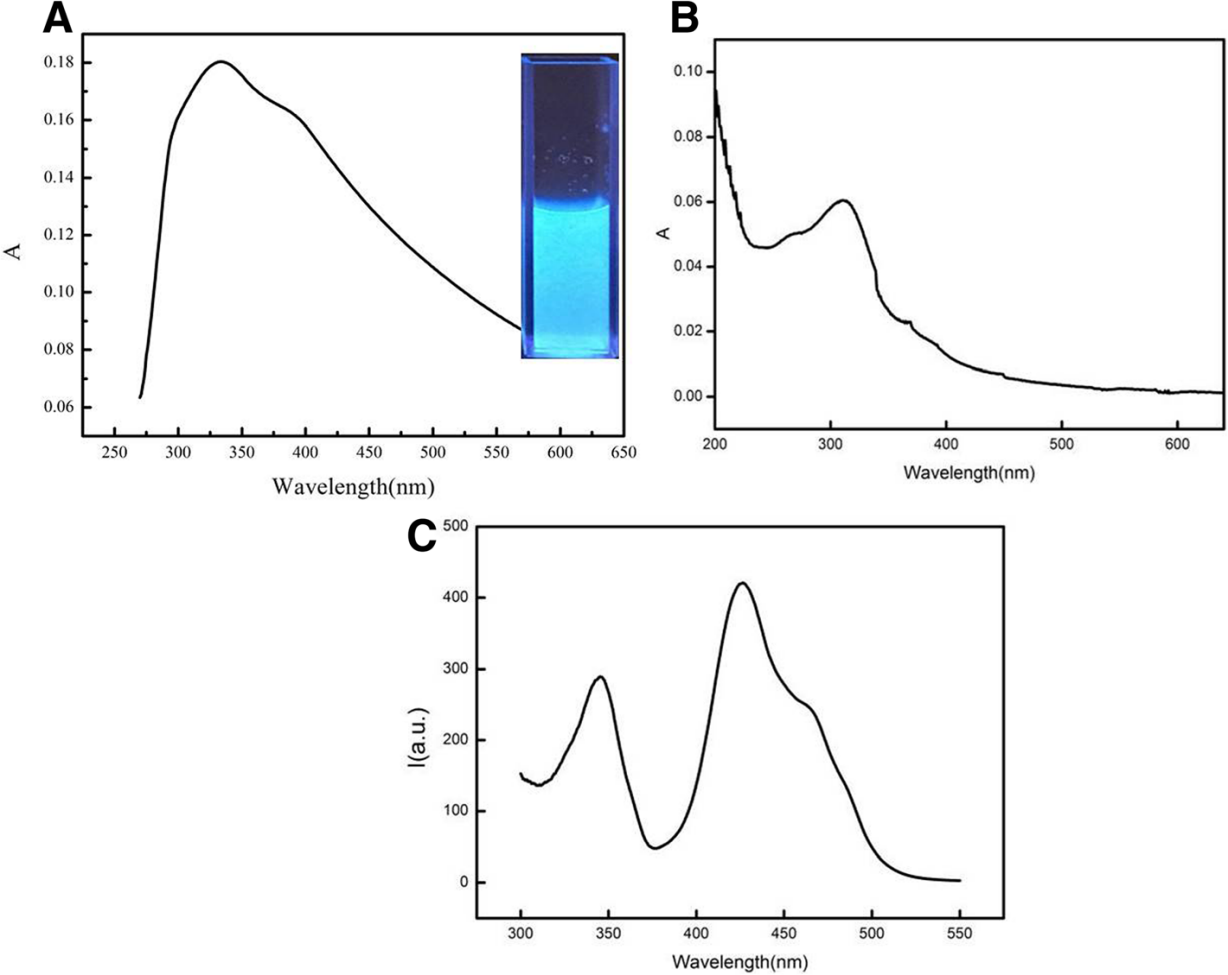
The UV-vis absorption and fluorescence spectrum of CdSe QDs/g-C_3_N_4_ conjugates

Fluorescence and UV-vis absorption spectra were obtained to evaluate the optical properties of CdSe QDs/g-C_3_N_4_ nanosheets. As shown in Fig. 3a, a large peak at approximately 334 nm was observed in the UV–vis absorption spectrum. Moreover, the fluorescence emission and excitation peaks were observed at 452 and 334 nm in the synchronous fluorescence spectroscopy in Fig. 3b and were associated with the emission fluorescence and ultraviolet light excitation of nanosheets. The emission peaks showed a shift compared with the pure g-C_3_N_4_ nanosheets at 14–16 nm (emission and excitation peaks were observed at 438 and 310 nm as presented in Fig. 3c), which could be ascribed to the FRET. The influence of excitation wavelengths on fluorescence intensities was also confirmed.

### Effect of pH to the Fluorescence of the CdSe QDs/g-C_3_N_4_ Nanosheet Conjugates

**Fig. 4:**
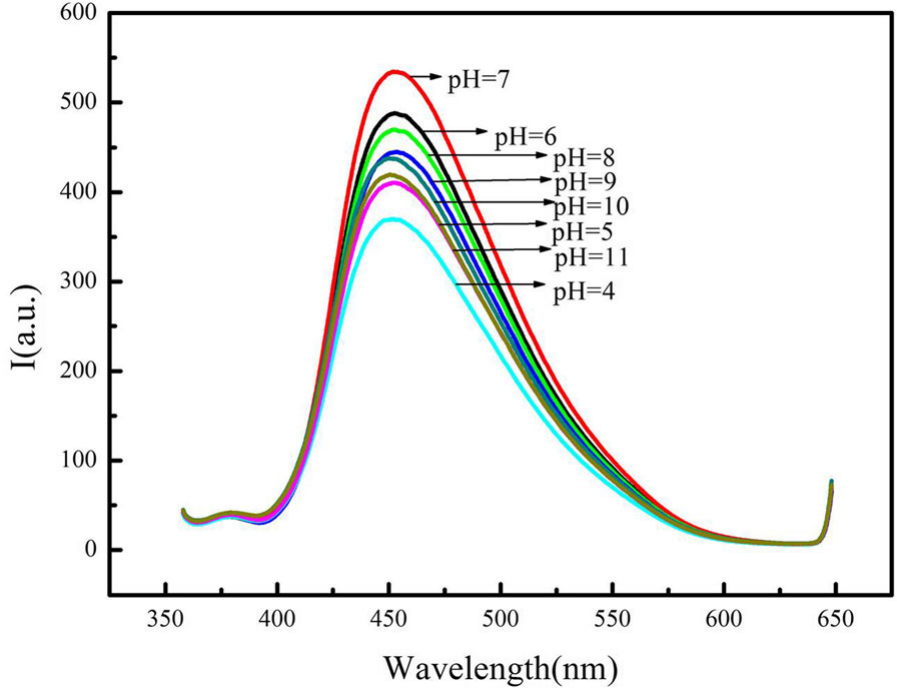
The effect of pH to the fluorescence of the CdSe QDs/g-C_3_N_4_ nanosheets conjugates

Figure 4 showed the fluorescence of the CdSe QDs/g-C_3_N_4_ nanosheet conjugates at different pH values. The pH value increased from 3 to 7 with the fluorescence intensity. However, the fluorescence intensity gradually decreased when the pH value varied increased from 7 to 10, which could be attributed to the effect of pH on the change in the surface charge owing to protonation–deprotonation due to the existence of amino groups in the structure of g-C_3_N_4_ nanosheets. In this study, the CdSe QDs/g-C_3_N_4_ nanosheet conjugates were conducted for the detection of Hg^2+^ ions, and the pH value of 7 was selected as the optimum pH value. The fluorescence emissions were measured at pH 7 containing different concentrations of NaCl to obtain the stability of the CdSe QDs/g-C_3_N_4_ nanosheet conjugates under high ionic strength circumstances. Only a slight change was observed under high ionic strength in the fluorescence intensities of the CdSe QDs/g-C_3_N_4_ nanosheet conjugates. The result showed that high ionic strength had minimal effects to the fluorescence intensities of the conjugates.

### Selectivity of CdSe QDs/g-C_3_N_4_ Nanosheet FRET System in Detecting Mercury Ion

**Fig. 5:**
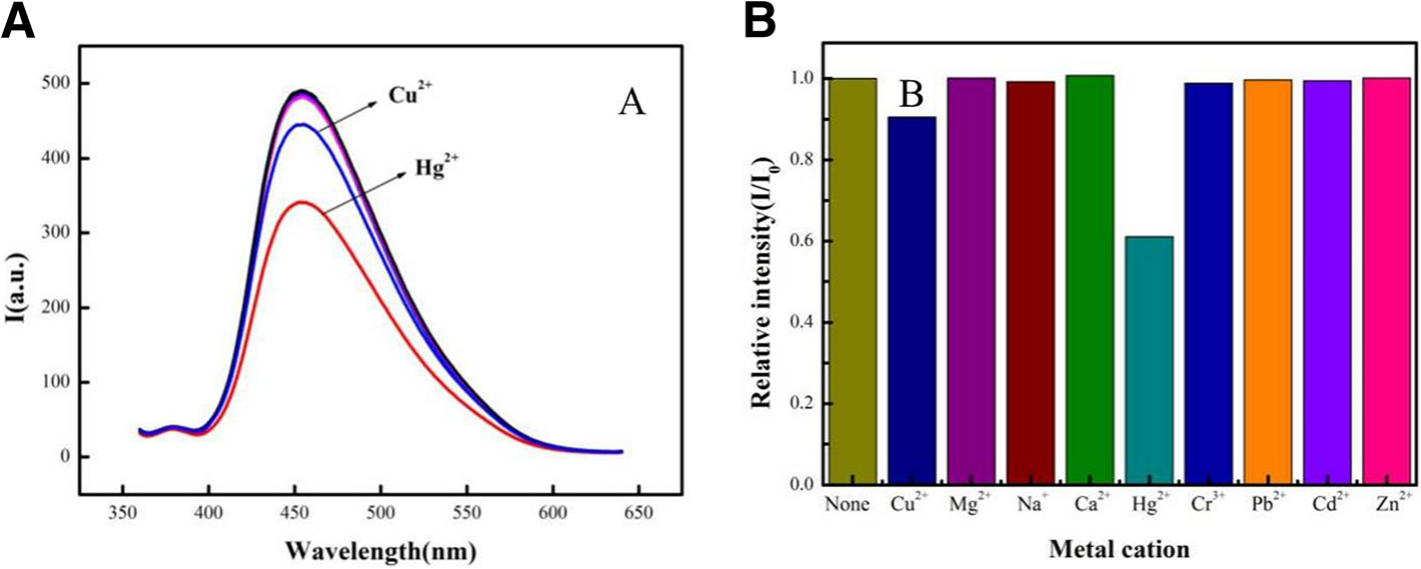
The selectivity experiments for the CdSe QDs/g-C_3_N_4_ nanosheets FRET sensor

Selectivity is an important parameter of a new sensing system. The selectivity of the CdSe QDs/g-C_3_N_4_ nanosheet FRET sensor was evaluated using various metal ions (e.g., Cu^2+^, Mg^2+^, Na^+^, Ca^2+^, Hg^2+^, Cr^3+^, Pb^2+^, Cd^2+^, and Zn^2+^); the results were shown in Fig. 5a. Compared with the blank sample without ions, the fluorescence ratio of Hg^2+^ increased obviously, while the fluorescence intensity of other metal ions changed slightly or remains the same. These results indicated that the FRET sensor showed more selectivity than the others (Fig. 5b). Thus, the CdSe QDs/g-C_3_N_4_ showed high selectivity toward Hg^2+^. This phenomenon was distinct in comparison with pure g-C_3_N_4_ nanosheet, which was selective for Cu^2+^ and Hg^2+^ [23, 24].

### Feasibility of the FRET Fluorescence Process in Detecting Hg^2+^

**Fig. 6:**
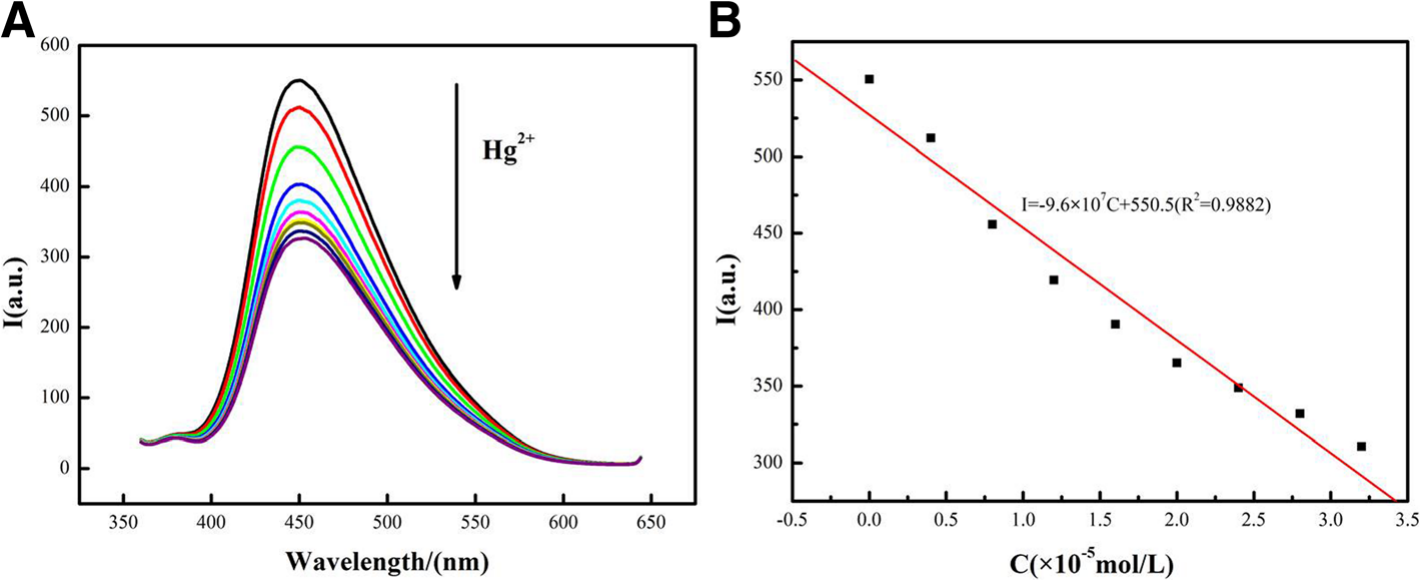
The sensing properties of the CdSe QDs/g-C_3_N_4_ nanosheets conjugates and the dependence of the fluorescence intensity to the concentration of Hg^2+^(C_Hg_^2+^: 1, 0 nM; 2, 4 nM; 3, 8 nM; 4, 12 nM; 5, 16 nM; 6, 20 nM; 7, 24 nM; 8, 28 nM; 9, 32 nM)

To study the practicability of the FRET sensor, the CdSe QDs/g-C_3_N_4_ nanosheet fluorescence detection of Hg^2+^ was performed. The presence of Hg^2+^ resulted in decreased fluorescence intensity as shown in Fig. 6, which illustrated that Hg^2+^ could effectively quench the FRET sensor. In order to study the sensitivity, the response of the sensor to different Hg^2+^ concentrations was further evaluated by fluorescence spectroscopy, and the results were shown in Fig. 6a. The fluorescence intensity of g-C_3_N_4_ nanosheets gradually decreased with increasing of Hg^2+^ concentrations. Figure 6b explained that the *I*/*I*_0_ was dependent on the concentration of Hg^2+^, where *I*_0_ and *I* were the fluorescence intensity in the absence and presence, respectively, of Hg^2+^. Moreover, the relation of *I*/*I*_0_ between concentrations of Hg^2+^ was linear, and the equation of linear regression was *I* = − 9.6 × 10^7^+ 550.5(*R*^2^ = 0.9882), as shown in the inset of Fig. 6b. Compared with recently reported luminescence methods, the proposed method had lower detection limit and higher sensitivity [25, 26]. The g-C_3_N_4_ nanosheets and CdSe QDs displayed no obvious quenching response to other metal ions apart from Hg^2+^, which suggested a relatively high selectivity for this method.

The other coexisting cations that affect the detection of mercury ion were detected as well. The response of the CdSe QDs/g-C_3_N_4_ nanosheet-based sensing system toward Hg^2+^ ion in the presence of alkali, alkaline earth, and other transition metal ions was shown in Table 1. The coexistence of most of the metal ions did not interfere with the binding of Hg^2+^, which indicated that the interference of these coexisting ions on the Hg^2+^ sensor was negligible.

**Table 1:** Effect of interfering species on the fluorescence intensity of FRET sensor solution under optimum conditions

Coexisting Substance	Concentraton/nmol/L	△F/F_0_ (%)
Ca^2+^	1.2	− 2.6
K^+^	0.2	+ 3.9
Li^+^	0.5	+ 3.5
Mg^2+^	1.0	+ 3.2
Al^3+^	2.0	− 1.8
Pb^2+^	3.0	+ 3.4
Mn^2+^	0.5	− 0.8
Ba^2+^	2.5	− 3.1
Cr^3+^	10	+ 2.6
Fe^3+^	8.0	+ 4.7
Fe^2+^	5.0	−2.8

△F = F − F_0_, where F and F_0_ are the fluorescence intensity in the presence and absence of interfering species

In addition, long-term stability is a superior property of sensors. The absorbance and the fluorescence during the continuous investigation every 3 days within 2 weeks indicated that the activity of CdSe QDs/g-C_3_N_4_ nanosheets remained above 92% of the initial efficiency though they were stored at ambient environment. The results indicated that the CdSe QDs/g-C_3_N_4_ nanosheets as FRET sensors had good long-term stability.

Compared with previous reports concerning fluorescence assays for Hg^2+^ (results are listed in Table 2), the CdSe QDs/g-C_3_N_4_ nanosheet fluorescence probe based on FRET with the concentration of Hg (II) in the range of 0–32 nmol/L at pH = 7 exhibited a limit of detection at 5.3 nmol/L. Thus, our method obtained a superior detection limit and linear range.

**Table 2:** The comparison of the previously reported and the present flurescence method for mercury ions

Fluorescence sensor	Detection limit (nM)	Solvent (medium)	Sensing mode	Reference
Silica particles	100	H_2_O	Ratiomeric	[27]
CdTe QDs	1.5	H_2_O	Fluorescence turn off	[28]
Tetraphenyborate anion	100	Membrane	Fluorescence turn off	[29]
Rhodamine derivative	20	H_2_O	Fluorescence turn on	[30]
CdS/ZnS QDs	40	H_2_O	Fluorescence turn off	[31]
Gold nanorods	500	H_2_O	Absorbance turn off	[32]
Mesoporous silica	1000	CH_3_CN	Fluorescence turn on	[33]
Mn:CdS/ZnS QDs	0.18	H_2_O	Fluorescence turn on	[34]
GO/ Au NPs	25	H_2_O	PET	[35]
CdTe QDs/rhodamine B	20.3	H_2_O	FRET	[36]
CdTe QDs/g-C_3_N_4_ nanosheets	5.3	H_2_O	FRET	This work

### Application of the FRET Sensor

The CdSe QDs/g-C_3_N_4_ nanosheets as a FRET sensor successfully provided a good platform for detecting Hg^2+^ in real samples because of their sensitivity and selectivity. Well, lake, and tap waters were selected as real samples for analysis in which the recovery of Hg^2+^ were in the range of 95.4–101.6% (Table 3). The relative standard deviation (RSD) of Hg^2+^ was in the range of 0.64–1.72%. The result stated clearly that the designed method can be efficiently used to detect Hg^2+^ in practical applications. The acceptable values of RSD and relative error confirmed the high sensitivity, high precision, and high reliability of the proposed FRET sensor for Hg^2+^ determination in practical applications.

**Table 3:** The results of the determination for mercury ions in practical samples

Samples	Found (nM)	Added (nM)	Found (nM, *n* = 3)	Recovery (%)
Well water	Not found	5	4.93	98.6
		10	9.54	95.4
		15	15.21	101.4
Tap water	Not found	5	5.06	101.2
		10	10.16	101.6
		15	15.18	101.2
Lake water	Not found	5	5.02	100.4
		10	10.13	101.3
		15	15.29	101.9

## Conclusions

A FRET-based system was developed for detecting Hg^2+^ within g-C_3_N_4_ nanosheets/CdSe QDs. The detection limit for Hg^2+^ ion was 5.3 nM, with a linear response ranging from 0 to 32 nM. The applicability of this sensor was demonstrated by measuring the content of Hg^2+^ in real samples. Given the long-term stability, low cost, and facile preparation of the CdSe QDs/g-C_3_N_4_ nanosheet conjugates, the fluorescence assay could be used as an environmental protection sensor. This strategy would provide an alternative approach for constructing FRET-based sensors for Hg^2+^ in aqueous media, including environmental and biological samples.

### Highlights

1. Fluorescence resonance energy transfer (FRET) system was constructed between CdTe quantum dots (QDs) (donor) and g-C_3_N_4_ (acceptor) in the presence of Hg^2+^ for the first time.
2. The nanocomposites of g-C_3_N_4_ supported by CdSe QDs (CdSe QDs /g-C_3_N_4_) were fabricated through a simple electrostatic interaction route in an aqueous solution.
3. The feasibility of the FRET system as a sensor was demonstrated for detecting Hg (II) in water solution. At pH 7, a linear relationship was observed between the quenched fluorescence intensity of the concentration of Hg (II) in the range of 0–32 nmol/L. The detection limit was 5.3 nmol/L.
4. The novel FRET-based fluorescence detection may provide an attractive assay platform for quantifying Hg^2+^ in complex water solutions.

## Abbreviations

FRET: Förster resonance energy transfer
FTIR: Fourier-transform infrared
UV–vis: Ultraviolet–visible
XPS: X-ray photoelectron spectroscopy
XRD: X-ray diffractometer
